# Alcohol Exposure Alters Mouse Lung Inflammation in Response to Inhaled Dust

**DOI:** 10.3390/nu4070695

**Published:** 2012-07-04

**Authors:** Michael L. McCaskill, Debra J. Romberger, Jane DeVasure, Jessica Boten, Joseph H. Sisson, Kristina L. Bailey, Jill A. Poole, Todd A. Wyatt

**Affiliations:** 1 Department of Environmental, Agricultural, and Occupational Health, College of Public Health, University of Nebraska Medical Center, Omaha, NE 68198, USA; Email: mmccaskill@unmc.edu (M.L.M.); dromberg@unmc.edu (D.J.R.); 2 VA Nebraska-Western Iowa Health Care System Research Service, Department of Veterans Affairs Medical Center, 4101 Woolworth Avenue, Omaha, NE 68105, USA; 3 Pulmonary, Critical Care, Sleep & Allergy Division, Department of Internal Medicine, 985300 Nebraska Medical Center, Omaha, NE 68198, USA; Email: jdevasure@unmc.edu (J.D.); Jessica.boten@unmc.edu (J.B.); jsisson@unmc.edu (J.H.S.); kbailey@unmc.edu (K.L.B.); japoole@unmc.edu (J.A.P.)

**Keywords:** alcohol, inflammation, mortality, organic dust

## Abstract

Alcohol exposure is associated with increased lung infections and decreased mucociliary clearance. Occupational workers exposed to dusts from concentrated animal feeding operations (CAFOs) are at risk for developing chronic inflammatory lung diseases. Agricultural worker co-exposure to alcohol and organic dust has been established, although little research has been conducted on the combination effects of alcohol and organic dusts on the lung. Previously, we have shown in a mouse model that exposure to hog dust extract (HDE) collected from a CAFO results in the activation of protein kinase C (PKC), elevated lavage fluid cytokines/chemokines including interleukin-6 (IL-6), and the development of significant lung pathology. Because alcohol blocks airway epithelial cell release of IL-6 *in vitro*, we hypothesized that alcohol exposure would alter mouse lung inflammatory responses to HDE. To test this hypothesis, C57BL/6 mice were fed 20% alcohol or water *ad libitum* for 6 weeks and treated with 12.5% HDE by intranasal inhalation method daily during the final three weeks. Bronchoalveolar lavage fluid (BALF), tracheas and lungs were collected. HDE stimulated a 2–4 fold increase in lung and tracheal PKCε (epsilon) activity in mice, but no such increase in PKCε activity was observed in dust-exposed mice fed alcohol. Similarly, alcohol-fed mice demonstrated significantly less IL-6 in lung lavage in response to dust than that observed in control mice instilled with HDE. TNFα levels were also inhibited in the alcohol and HDE-exposed mouse lung tissue as compared to the HDE only exposed group. HDE-induced lung inflammatory aggregates clearly present in the tissue from HDE only exposed animals were not visually detectable in the HDE/alcohol co-exposure group. Statistically significant weight reductions and 20% mortality were also observed in the mice co-exposed to HDE and alcohol. These data suggest that alcohol exposure depresses the ability of the lung to activate PKCε-dependent inflammatory pathways to environmental dust exposure. These data also define alcohol as an important co-exposure agent to consider in the study of inhalation injury responses.

## 1. Introduction

The pulmonary system is exposed to inhaled toxins and pathogens as a result of respiration. In turn, the pulmonary system has developed innate inflammatory responses to defend against these foreign invaders. A few of these inflammatory responses, such as inflammatory cell recruitment, can be modulated by alcohol exposure [1,2,3,4]. While the liver metabolizes most consumed alcohol, a significant percentage is exhaled unchanged [5]. This pulmonary exposure to alcohol can damage the bronchial epithelial layer of the lung [6,7], while impairing the lung’s ability to repair epithelial wounding via inhibition of the PKA pathway [8]. Additionally, alcohol inhibits the lipopolysaccharide (LPS)-induced inflammatory cytokine, interleukin-6 (IL-6), via the p38 ERK1/2 MAPK pathway [9]. It is also known that alcohol, at least in part, exerts its anti-inflammatory response via activation of the hypothalamic-pituitary-adrenal axis pathway resulting in increases of glucocorticoids [10]. Alcohol also interferes with proper inflammatory responses by disturbing the enzymatic processing of TNFα by TNFα Converting Enzyme (TACE) and abrogating TNFα/TACE function [11,12].

Animal husbandry is rife with occupational hazards. An emerging hazard is respiratory disease, including: occupation-related chronic bronchitis, chronic obstructive pulmonary disease (COPD), and exacerbation of asthma due to the inhalation of dust associated with swine husbandry [13]. This robust swine barn dust-induced inflammatory response is an essential component of the innate immune response. Historically, the trigger for the inflammatory response to swine dust exposure was attributed to endotoxins present in the dust [14]. However, recent publications describe an endotoxin-independent inflammatory effect in *in vivo* and *in vitro* swine barn dust exposures [15]. It is also well known that swine barn dust slows airway epithelial cell migration [16] and stimulates inflammatory cytokines IL-6 and IL-8 more potently than LPS [17,18,19]. 

Due to the changing socio-economic characteristics of American agricultural workers, old adages of the “healthy farmer” are dissipating. For instance, workers who are exposed to occupational organic dust also could be drinking significant volumes of alcohol. A Colorado State University farm-based occupational injury and alcohol consumption study [20] reports as high as 52% of the person days (8-h work day) surveyed involved two or more glasses of alcohol consumed. Another alcohol study conducted in rural Kentucky reports that 75% of those surveyed consumed alcohol to the point of intoxication at least three times a week for several years [21]. Midwestern states such as Nebraska and Iowa have some of the highest reported levels of rural alcohol usage (68%) and binge drinking (45%) for ages 18–25 in the United States [22].

Alcohol consumption leads to a muted pulmonary inflammatory response in alcohol-exposed rodents [23,24]. It is also known that hog confinement barn dust extract (HDE), when inhaled, leads to a severe pulmonary inflammatory response in C57BL/6 mice. We hypothesized that alcohol would depress the normative pro-inflammatory response observed in HDE-treated lungs. This study aims to evaluate *in vivo* effects on murine lung pathology and inflammatory cytokine response in alcohol-fed and HDE nasally instilled mice. 

## 2. Experimental Section

### 2.1. Mice

Female C57BL/6 mice were purchased from the National Cancer Institute (Frederick, MD) at 7–8 weeks of age and under standard housing conditions. Mice were acclimated to the AAALAC-certified facility at the University of Nebraska Medical Center for 1 week before the start of exposure and received standard rodent chow *ad libitum* for the entire course of the study. Mice were monitored daily and weighed weekly. All experimental protocols were reviewed and approved by the Institutional Animal Care and Use Committee of the Omaha Veterans Affairs Medical Center. All protocols conformed to the *Guide for the Care and Use of Laboratory Animals* of the National Institutes of Health.

### 2.2. Organic Dust Exposure

Organic dust was collected from two different confinement swine barns containing at least 500 individual animals. The organic dust was then made into an extract (HDE) by methods previously described [25]. Mice received saline or HDE by an intranasal exposure method as previously established using 12.5% HDE as an optimal concentration for eliciting lung inflammation [26]. Mice received daily nasal instillations of 12.5% HDE for 3 weeks prior to sacrifice. 

### 2.3. Alcohol Feeding

Mice receiving alcohol were given increasing concentrations of alcohol in water over a 2-week period until the target concentration of 20% was reached using the Meadows-Cook model [27,28]. Mice in the alcohol group were given 5% alcohol (wt/vol) to drink *ad libitum* and scaled up over two weeks to 20% alcohol (wt/vol) for 6 weeks [30]. Mice in the matched control group were given water from the same source without alcohol. The alcohol exposure model utilized is effective in achieving average blood alcohol concentration (BAC) levels of 149 ± 47 mg/dL in the alcohol-fed mice [29].

### 2.4. Bronchoalveolar Lavage (BAL)

After the instillation period, mice were euthanized by intraperitoneal injection of 50 mg/kg body weight of pentobarbital Nembutol (Abbott Labs, Chicago, IL, USA). The trachea was exposed and a cannula inserted just below the larynx. The proximal end of the trachea was held around the cannula with forceps while 1.0 mL of sterile PBS (Gibco, Grand Island, NY, USA) was instilled into the lungs and recovered by aspiration. A total of 3.0 mL was introduced to the lungs. The BAL fluid was centrifuged at 250 *g* to collect cells. The supernatant from the first milliliter of BAL fluid recovered was frozen at −80 °C to later test for cytokines. Cells from all 3 mL were resuspended, pooled and spun onto slides with a Cytopro Cytocentrifuge (Wescor Inc. Logan, UT, USA) and stained with DiffQuik (Dade Behring, Newark, DE, USA). Counts of the cells determined the differential ratio of cell types in 200 cells per slide per mouse. Tracheae were removed, after BAL collection, and trachea epithelial cells were harvested from the trachea by scraping and placed in 500 µL of Tris-HCl (50 mM, pH 7.4) lysis buffer with protease inhibitors as previously described [30]. Trachea epithelial cells were then homogenized, sonicated and spun at 10,000 *g* at 4 °C in an ultracentrifuge. These samples were then flash frozen in liquid nitrogen and stored at −80 °C until assayed for PKC activity.

### 2.5. Lung Histology

After BAL, whole lungs were excised and inflated to 10 cm H_2_O pressure with 10% formalin (Sigma-Aldrich, St. Louis, MO, USA) solution to preserve pulmonary architecture. The lungs were embedded in paraffin and sections (4–5 µm) were cut and stained with hematoxylin and eosin (Sigma-Aldrich, St. Louis, MO, USA). 

### 2.6. PKC Isoform Assay

From the previously frozen tracheal epithelial samples, the cell supernatant was removed (cytosolic fraction) and the cell pellet resuspended in cell lysis buffer [31] containing 0.01% Triton X-100 and sonicated again (particulate fraction). PKC isoform activity was determined in these tracheal cytosolic and particulate samples similar to methods described previously [31]. Tracheal epithelial cells have functional α, β, δ, ε, and ζ PKC isoforms [32]. To measure specific PKC isoform activity, 24 μg/mL PMA, 30 mM dithiotreitol, 150 μM ATP, 45 mM Mg-acetate, PKC isoform-specific substrate peptide, and 10 μCi/mL [γ-^32^P]-ATP were mixed in a Tris-HCl buffer (pH 7.5). Samples were cooled to 4 °C. Samples (20 μL) were added to 40 μL of the reaction mix and incubated for 15 min at 30 °C. This mixture (60 μL) was then spotted onto P-81 phosphocellulose papers (Whatman, Clinton, NJ, USA) to halt incubation, and papers were subsequently washed 5 times in 75 mM phosphoric acid for 5 min, washed once in 100% alcohol for 1 min, dried, and counted in nonaqueous scintillant (National Diagnostics, Atlanta GA, USA). PKC activity was expressed in relation to the total amount of cellular protein assayed as picomoles of phosphate incorporated per minutes per milligram. Similarly, PKA activity was determined as previously described [33].

### 2.7. Cytokine Assays

BALF was collected and assayed after treatment for the concentration of interleukins released using a sandwich ELISA. IL-6 and IL-8 levels in BALF were assayed in duplicate and quantified by sandwich ELISA according to the manufacturer’s instructions (R&D Systems, Minneapolis, MN, USA) with minimum sensitivities of 1.6 and 2.0 pg/mL, respectively. Cytokine/chemokine secretion is reported as concentration (pg/mL), respectively. 

For the TNFα-specific ELISA, assay conditions were as described above with the following exceptions: plates were coated with monoclonal anti-mouse TNFα at 2 µg/mL, the secondary “bridge” antibody was biotinylated (rabbit) anti-mouse TNFα at 200 ng/mL, which was detected with steptavidin-HRP (1:200). The enzyme substrate was a two-part commercially available kit (H_2_O_2_ and tetramethylbenzidine, R&D Systems). For all ELISAs, the reaction was terminated with 27.5 µL/well of 8 M sulfuric acid and plates were read at 490 nm or 450 nm in an automated ELISA reader (Dynex Technologies, Chantilly, VA. Results are expressed as pg of cytokine/mL.

### 2.8. Lung Slice Protocol

Female C57Bl/6 mice, between 7 and 9 weeks old, were sacrificed by intraperitoneal injection of 0.3 mL of sodium pentobarbital. The trachea was cannulated with an intravenous (IV) catheter tube with two input ports (20G Intima; Becton Dickinson) and secured with suture thread (4–0). A syringe filled with 3 mL of air was attached to one port while the other port was closed. The lungs were gently reinflated to approximate their total lung capacity by injecting 1.5 mL of air. Lungs were allowed to deflate after which a syringe filled with a warm (37 °C) solution of 2% agarose (type VII or VII-A: low gelling temperature; Invitrogen, Carlsbad, CA, USA) in sHBSS was attached to the second port of the catheter. Immediately after agarose inflation, the lungs were washed with ice-cold sHBSS, and the mouse carcass was cooled at 4 °C for 30–45 min. After the cool down period the lungs and heart were removed and placed in sHBSS (4 °C) and cooled for an additional 30 min–1 h to ensure the complete gelling of the agarose. A single lung lobe was removed from the respiratory tree by cutting the main bronchus. The lobe was trimmed near the bronchus to create a flat surface. The trimmed lobe was then held in place by tissue adhesive to the mounting block of the vibratome of the Electron Microscopy Sciences Tissue slicer (OTS 4500). The mounted lobe was submerged in a bath of HBSS maintained at 4 °C. The lung lobe was sectioned into slices 140 μm thick starting at the lung periphery. Sections were collected and transferred individually to wells of a 24-well plate containing DMEM supplemented with antibiotics fetal calf serum and anti-mycotics. The slices were kept at 37 °C and 10% CO_2_ for at least 5 days prior to treatment. Twenty-four hours prior to treatment, media was changed to serum free to reduce interference with cytokine assays. Lung slices were pretreated for 24 h with alcohol at 80 mM prior to 5% HDE exposure. The slice experiments were performed in triplicate. Each data point represents *n* = 3 or more different mice using multiple lung slices from different lobes of the lung. 

### 2.9. Statistical Analyses

Results presented in this manuscript are expressed as the mean ± SEM of the number of animals in each group. The statistical differences between the various group means were determined using the one-way ANOVA with Bonferroni post-test (Graphpad Prism, San Diego, CA, USA). A probability of less than 0.05 was accepted as significant.

## 3. Results

### 3.1. Body Weights and Mortality

**Figure 1:**
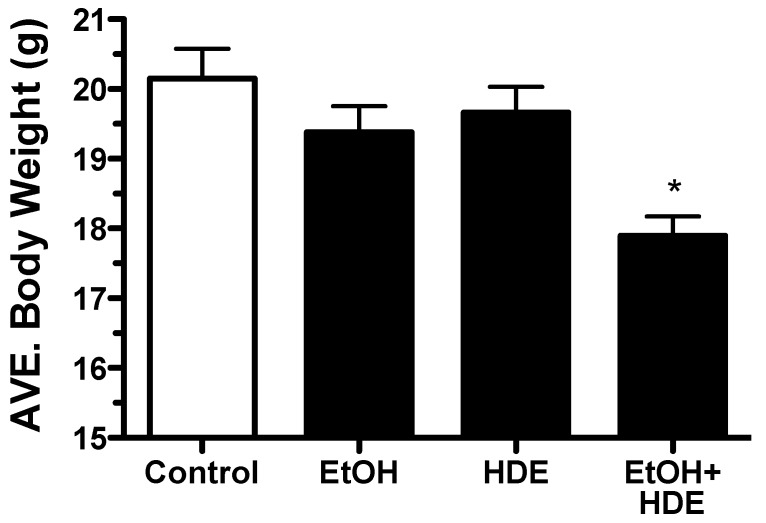
C57BL/6 Mice were fed alcohol (EtOH) for 6 weeks and were exposed to 12.5% hog barn dust extract (HDE) intranasally for 3 weeks (*n* = 5). Body weights of the alcohol + HDE group were statistically lower as compared to control group weights (17.9/20.1 g) (* *p* < 0.05 *vs*. control ANOVA, Bonferroni post-test).

C57BL/6 mice were fed alcohol *ad libitum* for 6 weeks and at the 3-week halfway point, some mice (protocol designated) were exposed to 12.5% HDE intranasally for the remainder of the 6-week protocol. At the end of the 6-week protocol, it was observed that the body weights of mice in the combination alcohol + HDE group were statistically lower as compared to the control group weights (*p* < 0.001, 17.9/20.1 g) (Figure 1). Additionally, 20% of the co-exposed alcohol + HDE group died and collectively lost weight due to the treatment. The cause of the morbidity was unidentified (Figure 1). 

### 3.2. Inflammatory Cells

**Figure 2:**
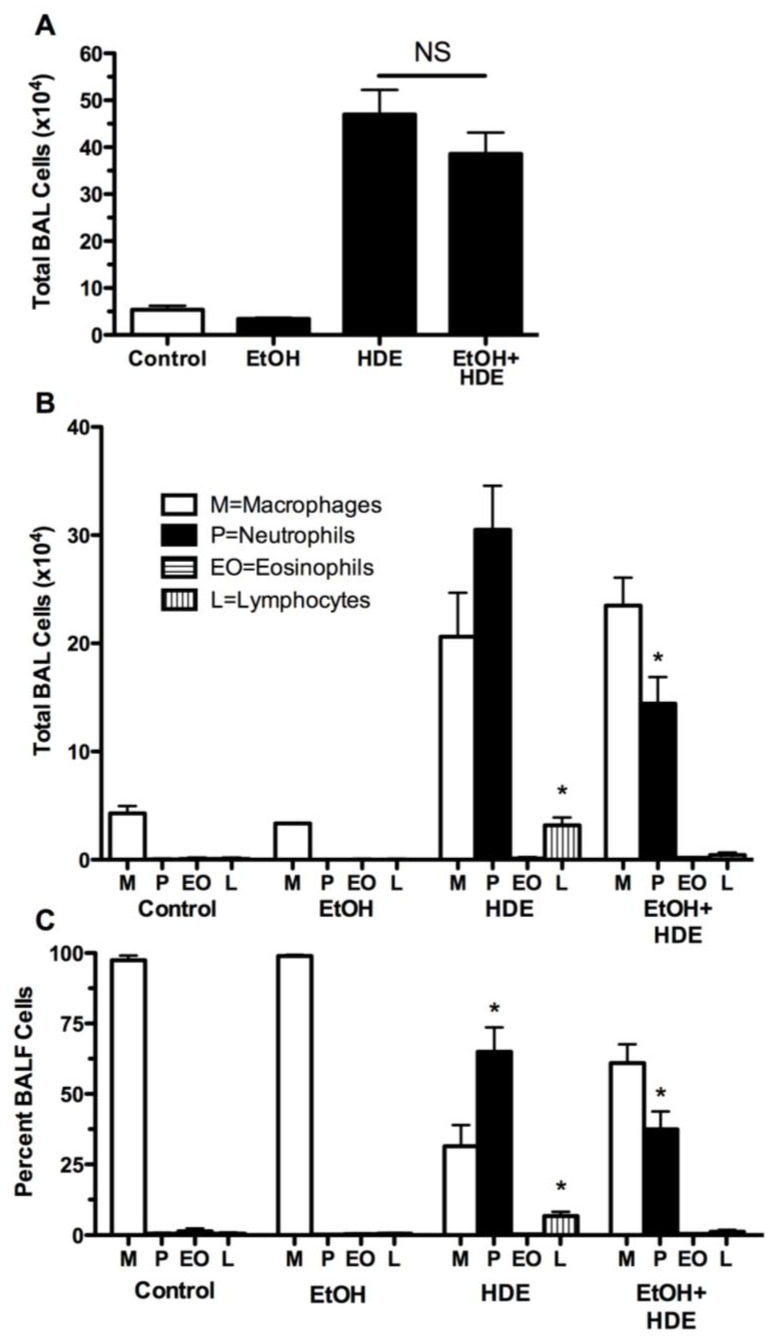
C57BL/6 Mice were fed alcohol (EtOH) for 6 weeks and were exposed to 12.5% HDE intranasally for 3 weeks (*n* = 5). (**A**) Total cells counted in BALF: Total cellularity collected in the bronchoalveolar lavage fluid (BALF) increased nearly 10-fold in the HDE and alcohol + HDE groups however there is no statistical difference between the HDE and co-exposure group. (**B**) Total cells counted in BALF per cell type: Inflammatory cells quantified from BALF detailed a statistical increase in neutrophils in the HDE group, which is followed by a reduction in neutrophils in the alcohol + HDE group. Inflammatory cells quantified from BALF detailed a difference in distribution between alcohol + HDE and the HDE group alone. (**C**) Percentage of each type of cells coutnted: Neutrophils increased from less than 1% in the control to 65% in the HDE group and back down to 37% in the alcohol + HDE group. (* *p* < 0.05 *vs*. control ANOVA, Bonferroni post-test) (M = macrophages, P = neutrophils, EO = eosinophils, L = lymphocytes).

Twenty-four hours after the final HDE instillation BAL fluid was collected, the cells were counted and the total cell counts were not significantly different between the combined alcohol + HDE group and the HDE alone group. However, the distribution of cell types differed based on treatment groups. The percentage of neutrophils increased from less than 1% in the control group to 65% in the HDE group. However, a significant decrease (37% reduction) in BAL neutrophils was observed in the alcohol + HDE group as compared to the HDE group (Figure 2).

### 3.3. Cytokines

**Figure 3:**
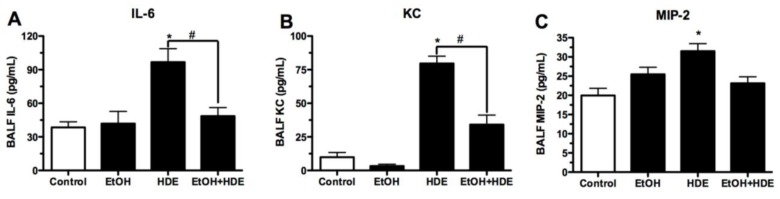
C57BL/6 Mice were fed alcohol (EtOH) for 6 weeks and were exposed to 12.5% HDE intranasally for 3 weeks (*n* = 5). BAL fluid was collected from these mice and cytokine levels were quantified via ELISA. (**A**) IL-6 was ~50% less in the alcohol + HDE group as opposed to the HDE only group (46.8/96.1 pg/mL);(**B**) KC quantification detailed a statistically significant reduction in the alcohol + HDE group compared to HDE only group (34.1/79.6 pg/mL); (**C**) MIP-2 quantification detailed a statistically significant reduction in the alcohol + HDE *vs*. the HDE only group (23.1/31.5 pg/mL) (* *p* < 0.05 *vs*. control ANOVA, Bonferroni post-test; ^#^*p* < 0.05 *vs*. HDE, ANOVA, Bonferroni post-test).

**Figure 4:**
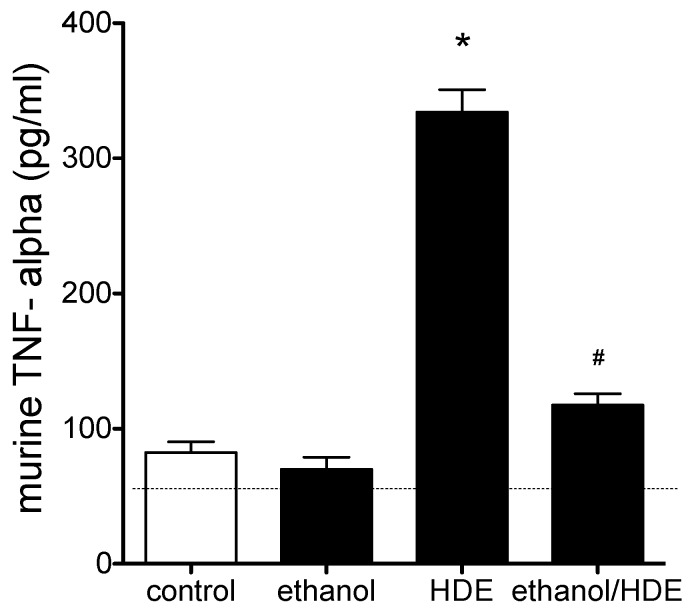
C57BL/6 Mice were fed alcohol (EtOH) for 6 weeks and were exposed to 12.5% HDE intranasally for 3 weeks (*n* = 5). Cytokine in the BAL fluid collected from these mice was quantified by ELISA. HDE instillation elevated TNF release more than 3-fold *vs*. control (* *p* < 0.05 *vs*. control, ANOVA, Bonferroni post-test). TNF-α levels were reduced by 65% in the alcohol + HDE group compared to the HDE only group (117.6/334.1 pg/mL). (^#^*p* < 0.05 *vs*. HDE, ANOVA, Bonferroni post-test).

After the completion of the 6-week alcohol feeding and HDE instillation protocol, cytokines in the BAL fluid were quantified. We observed approximately a 50% reduction in BAL IL-6 levels in the alcohol + HDE exposed group as compared to the HDE only group (*p* < 0.01, 46.8/96.1 pg/mL; Figure 3). Similar patterns were seen with KC (34.1/79.6 pg/mL) and MIP-2 (23.1/31.5 pg/mL) (Figure 3). Alcohol treatment attenuated HDE-induced TNFα stimulation by 65% (117.6/334.1 pg/mL) as compared to the HDE only group (Figure 4).

### 3.4. Protein Kinase Activity

**Figure 5:**
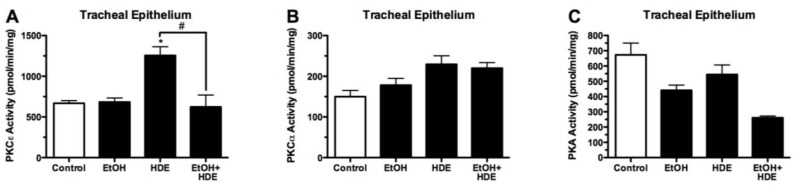
C57BL/6 Mice were fed alcohol (EtOH) for 6 weeks and were exposed to 12.5% HDE intranasally for 3 weeks (*n* = 5). Protein kinase activity was quantified from the tracheal epithelium removed from these mice. (**A**) PKCε activity in the tracheal epithelial cells of the alcohol + HDE group was statistically significantly lower as compared to the HDE only group (622.2/1255.5 pmol/mg/min) (^#^*p* < 0.05); (**B**) PKCα activity in tracheal epithelial cells was unchanged in the epithelial cells unchanged in the treatment groups; **(C**) PKA activity in the tracheal epithelial cells of the alcohol + HDE treated mice was ~50% lower than the HDE only treated mice (261.1/544.6 pmol/mg/min) (^#^*p* < 0.05).

**Figure 6:**
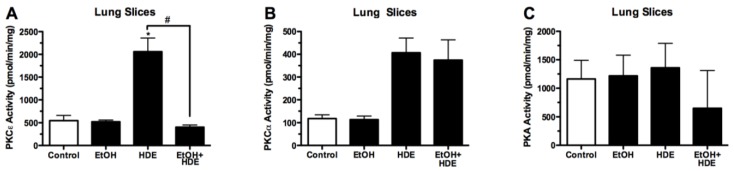
C57BL/6 Mice were fed alcohol (EtOH) for 6 weeks and were exposed to 12.5% HDE intranasally for 3 weeks (*n*=5). Protein kinase activity was quantified from the *ex-vivo* lung tissue removed from C57Bl/6 mice. (**A**) PKCε activity in the lung slices of the alcohol+HDE treated tissue was ~80% less than the activity of observed in the HDE only group (404.1/2058.5 pmol/mg/min);(**B**) PKCα activity in the lung slices of C57Bl/6 mice treated with alcohol+HDE and HDE only were both statistically higher than the control;(**C**) PKA activity was unchanged between treatment groups (* ^#^*p*< 0.05 *vs*. control ANOVA, Bonferroni post-test).

Protein kinase activity was quantified from the tracheal epithelial cells removed from alcohol-fed and HDE-instilled mice. Protein kinase C epsilon (PKCε) activity in the tracheal epithelial cells of the alcohol + HDE exposed mice was ~50% less than activity in the HDE only group (622.2/1255.5 pmol/mg/min) (Figure 5). PKC-ε activity in the lung slices of alcohol + HDE exposed mice was ~80% less than the activity in the HDE only group (404.1/2058.5 pmol/mg/min) (Figure 6). The protein kinase A activity in the tracheal epithelial cells of alcohol + HDE exposed mice was ~52% lower than the HDE only exposed mice (261.1/544.6 pmol/mg/min) (Figure 5). 

### 3.5. Lung Histology

**Figure 7:**
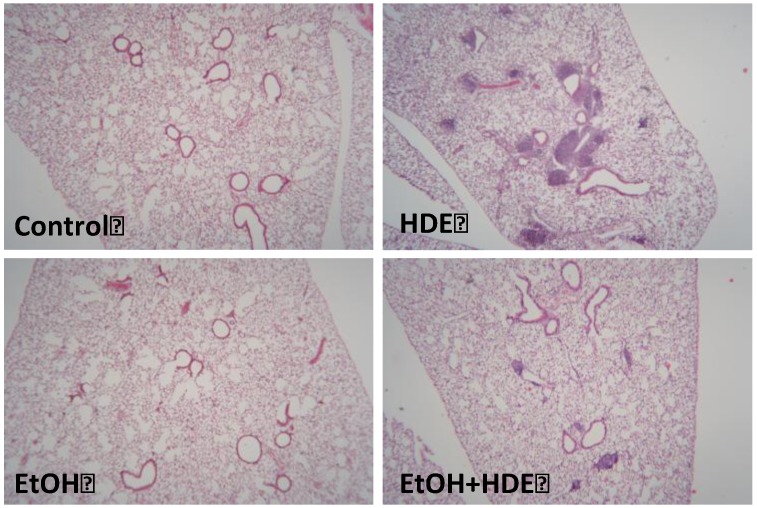
C57BL/6 Mice were fed alcohol (EtOH) for 6 weeks and were exposed to 12.5% HDE intranasally for 3 weeks (*n* = 5). Lung sections from H&E stained slides revealed upon histological evaluation that HDE exposed C57BL/6 mice exhibited the highest levels of peribronchial inflammation. The alcohol + HDE exposed group showed a marked reduction in HDE-induced mononuclear cellular aggregates.

At the completion of the treatment protocol and following BAL collection, lung tissue was stained with hematoxalyn and eosin (H&E) and underwent qualitative analysis. The mice exposed to HDE had noticeable mononuclear cellular aggregates indicative of high levels of peribroncholar inflammation. The lungs of mice exposed to 6 weeks of 20% alcohol *ad libitum* and 12.5% HDE nasally instilled for 3 weeks showed marked reduction of mononuclear cellular aggregates (Figure 7). 

## 4. Discussion

**Figure 8:**
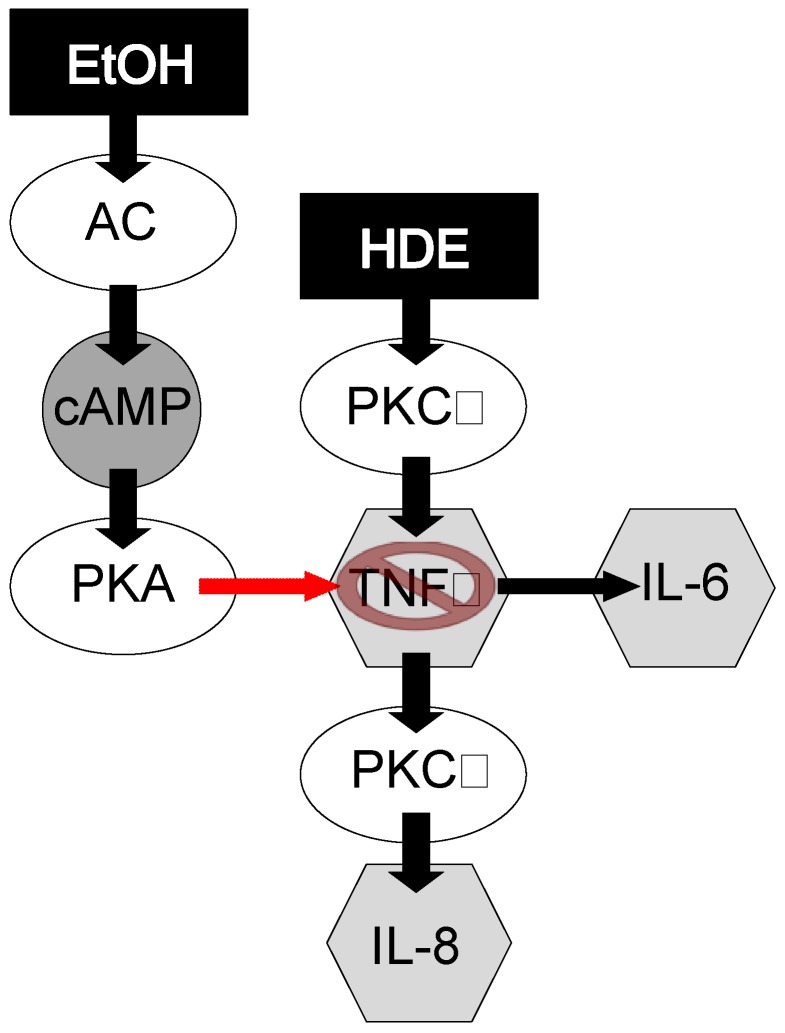
Proposed mechanism by which alcohol exposure ablates HDE-induced inflammation.

In an inflammatory state induced by HDE inhalation, it has been observed by our group that the HDE-induced inflammatory insult is followed by rapid PKCα activity, which stimulates TNFα release that induces IL-6 and PKCε stimulation concluding with a subsequent increase of IL-8 activity [17,34] (Figure 8). The observed data presented in this manuscript parallels this previously published sequential signaling. In accord with previously published manuscripts [26,35,36], HDE exposure in our experiments caused robust statistical increases in inflammatory cell recruitment, mediator release, protein kinase C activity, and lung pathology development. In the HDE-only exposed group we observed increases in levels of IL-6, IL-8, TNFα, PKCε and PKA, all of which promote the inflammatory response. However, when alcohol was fed to HDE-exposed mice via the Meadows & Cook model, much of the HDE-induced inflammatory response was muted, or in the case of some cytokines, completely abrogated. These data suggest that the consumption of alcohol interrupts the inflammatory process by inhibiting signaling such as KC, MIP-2 (IL-8) and IL-6, along with down regulating the activity of PKCε in the co-exposed HDE + alcohol group. Alcohol-mediated protein kinase inhibition has been observed and cited in prior *in vitro*-based publications [37,38]. Our current data show that PKA inhibition by alcohol is also present in the *in vivo* model. Interestingly enough, HDE exposure statistically increased PKCα levels in the lung tissues and tracheal epithelial cells of mice, however unlike PKCε and PKA, co-administration of alcohol and HDE did not reduce PKCα levels. This observation is key because PKCα has been shown to stimulate the cascade of signaling that regulate IL-6 release, PKCε and subsequent IL-8 release [17]. Our data shows that alcohol consumption inhibits this PKCα initiated inflammatory response, and the lack of a subsequent KC/MIP-2 (IL-8) protein increase, under pro-inflammatory conditions, led us to conclude that the signaling disruption may be occurring between the PKCα activation and IL-6 vitiation. In our proposed sequential model [17] (Figure 8), TNFα represents an intermediate in the pathway between PKCα and chemokine release.

TNFα is a cytokine that mediates inflammatory responses via the NF-kB pathway. Previously, our group have shown that TNFα protein stimulates PKCε but not PKCα [17], and subsequently TNFα stimulates IL-6 and IL-8 (KC/MIP-2) [19], which in turn induces neutrophil migration to the site of inflammation [39]. Even though total levels of BALF cells were statistically indifferent, in our data, neutrophil levels in the BALF were statistically lower as a proportion of total inflammatory cell counts in the alcohol + HDE group as compared to the HDE group. This is important because neutrophils are the first of the inflammatory cells to migrate towards the site of inflammation or injury [40], and these neutrophils are called to the site of inflammation by the potent neutrophil chemokine, IL-8 (KC/MIP) [41]. With alcohol attenuating HDE-stimulated release of IL-8 mouse homologues (KC/MIP-2), a reduction of neutrophils in BALF is an expected response [41,42]. 

Peribronchiolar inflammatory cell aggregates normally generated by the mouse lung’s response to HDE were not as prevalent in the alcohol + HDE-exposed mice. This lack of cellular aggregates is likely mediated by decreased neutrophil activity. Neutrophils themselves are potent proinflammatory mediators [43], and the effect of the absence of neutrophils in an *in vivo* model under the influence of highly inflammatory insult such as HDE was previously unknown. 

In our study, we observed 20% (2/10) mouse mortality. Even though *n* = 10 is a small sample size, the statistical reduction of body weights in the co-exposed group shows that there was a level of general malaise in the group, which was unexpected and led us to speculate that alcohol’s interference in the function of TNFα in a high inflammatory environment could be a reason for these deleterious results observed in the alcohol + HDE co-exposed group. Alcohol has been shown to inhibit neutrophil cytokines including TNFα release [44], IL-8, and NF-kB activation [45]. According to Taieb *et al*. 2002, this inhibition is, in part, attributed to alcohol’s ability to inhibit the expression and function of TNF converting enzyme (TACE) [44]. TACE is an enzyme that proteolytically cleaves membrane bound TNFα allowing for soluble TNFα to be active. Pulmonary epithelial cells cannot mount a proper TNFα-mediated response to an inflammatory mediator such as HDE without soluble TNFα [46]. These data have shown an inhibition of said TNFα-mediated response by alcohol in the presence of a robust inflammatory initiator. As a result under pro-inflammatory pressures, the inhibition of TACE will lead to increased levels of membrane-bound TNFα which could potentially stimulate some TNFα-mediated responses [47], not the least of which could be cachexia [48]. We acknowledge that serum TNFα levels, liver TNFα levels and an autopic pathological evaluation would be useful in elucidating the unexpected morbidity and mortality. Studies are being conducted to further characterize observations detailed in this manuscript. However with the weight loss and mortality data we have currently, it is possible to postulate that the mice in the co-exposed group were entering various levels of cachexia. This may seem to be contradictory to the TNFα data presented, however it has been observed that elevated levels of TNFα resulting from chronic alcohol can be attenuated with acute larger doses of alcohol [49]. Concurrently it is well documented that alcohol reflux within the lungs due to the exhalation and condensation of alcohol onto the muscosal layer of the upper airways exposes the lung epithelial layer to concentrations of alcohol beyond what the lungs would be exposed to systemically. However, unlike the liver, the lung does not have robust stores of enzymes primed to metabolize compounds to allow for rapid excretion. As a result, direct pulmonary epithelium exposure is prolonged, leading to possibly a more acute response to alcohol exposure locally in the lung even under systemic chronic alcohol pressures. This acute response in a chronic exposure environment may be what is mediating the attenuated TNFα observed in the lung tissue. 

Based on the published data and our data presented here, it is reasonable to propose that alcohol interferes with the proper HDE-induced inflammatory response possibly through the TACE/TNFα pathway, leading to reduced IL-6, PKCε, KC/MIP-2 activity, improper infiltrate clearance, weight loss, and mortality. Studies are currently underway to investigate the effect of alcohol on TNFα/TACE function during HDE exposure. Ultimately, these data relay the importance of a properly controlled proper inflammatory response, for when hog dust-induced inflammation is attenuated by alcohol consumption, C57Bl/6 mice have morbidity and mortality of which the etiology is currently unknown. 

## 5. Conclusion

In conclusion, an over-stimulated inflammatory response can lead to any number of inflammatory lung diseases such as chronic bronchitis, COPD, or asthma. In these conditions controlling inflammation is a primary goal. However, under the constant pressure of a potent inflammatory mediator such as organic dust from a swine confinement facility, alcohol’s inflammatory suppression led to a deleterious outcome in our mouse model. Future studies will be conducted to investigate the nature and severity of the systematic failure which led to increased morbidity and mortality observed in alcohol and confinement barn dust (HDE) co-exposure models.
